# Experimental study of centrifugal pumping of magnetorheological fluid

**DOI:** 10.1177/1045389X251344476

**Published:** 2025-06-14

**Authors:** Tim Dumais, Jean-Sébastien Plante, David Rancourt

**Affiliations:** 1 Interdisciplinary Institute for Technological Innovation (3IT), Faculty of Engineering, Université de Sherbrooke, Sherbrooke, QC, Canada; 2 Exonetik, Sherbrooke, QC, Canada

**Keywords:** Magnetorheological, clutch, pump, centrifugal, sedimentation, durability

## Abstract

This paper studies the potential of using a centrifugal pump to handle magnetorheological fluid with the ultimate goal of integrating such a pump directly into a magnetorheological clutch to increase MR actuator life and stabilize performance over time. A centrifugal pump design is proposed, and two prototype sizes are constructed. Experimental characterization of the pumps prototypes reveals that, even with a magnetorheological fluid containing 40% V/V of ferromagnetic microparticles, the standard scaling laws for centrifugal pumps provide accurate predictions of performance with changes in rotation speed. However, the results showed that these laws do not fully account for geometric effects when scaling the pump size due to differences in impeller performance. The results also show that a centrifugal pump of 46 mm in diameter can effectively circulate magnetorheological fluid at a flow rate of 90 mL/min per shear interface in a commercial MR clutch.

## 1. Introduction

### 1.1. Motivation

The aerospace industry is transitioning toward more electric aircraft (MEA) to reduce environmental impact while simultaneously attempting to lower maintenance requirements and costs (Mazzoleni et al., 2021). In this context, there is a demand for novel electric actuators capable of high bandwidth, high power density, and full backdrivability to replace existing hydraulic actuators used for flight control.

Most conventional electromechanical actuators that could be considered as direct-replacement for hydraulics use gears or screws, posing a fundamental safety risk due to possible jamming of metal-to-metal contacts (Hussain et al., 2018). Jamming is an unacceptable failure mode in primary flight control systems actuator as it is a single point, catastrophic failure.

Magnetorheological (MR) actuators provide a solution to jamming since loads are transmitted by a fluid interface, without direct metal-to-metal contact. This design ensures that in the event of a critical failure of the MR actuator, the pilot or auxiliary systems can backdrive the faulty actuator.

**Figure 1.**
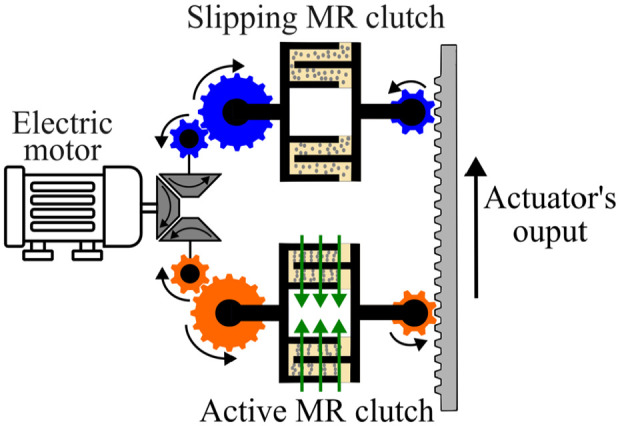
Working principle diagram of the MR actuator with continuous slip clutch. The green arrows represent the magnetic field.

MR actuators typically consist of a motor and two antagonistic MR clutches continuously held in slippage as shown in Figure 1. Torque output can be control with high precision and bandwidth (<15 Hz; Kavlicoglu et al., 2007) control can be achieved by modulating the magnetic field, and thus, viscosity of the magnetorheological fluid (MRF).

Due to their unique potential and capabilities, magnetorheological (MR) clutches and brakes have been the focus of extensive research in recent years. New designs have been developed to enhance performance and controllability (Chen et al., 2022; Kikuchi et al., 2024; Park et al., 2021; Pisetskiy and Kermani, 2021; Shiao and Kantipudi, 2022; Wu et al., 2023a, 2023b; Xiong and Huang, 2023; Zhang et al., 2024). Numerous applications have also been explored, including automotive systems (Duan et al., 2023; Park et al., 2024; Turcotte et al., 2022; Wang and Choi, 2021; Yang et al., 2023, 2024; Zhao et al., 2024), human support devices such as prostheses and exoskeletons (de Andrade et al., 2021, 2023; Morimura et al., 2022; Veronneau et al., 2022), and haptic technologies (Ikeda et al., 2023; Kikuchi et al., 2021; Lhommeau et al., 2023; Takano et al., 2022). In the context of primary flight controls, a study of Chouinard et al. (2015) have demonstrated the significant potential of MR actuators, although practical challenges arise:

Practical Challenge 1—Finite lifetime As introduced by Carlson (2002), MR fluid life is defined as the Lifetime Dissipated Energy (LDE) for a given volume of fluid. Furthermore, in high power applications, the slippage operation of MR actuators leads to temperature increase which can accelerate degradation (Wiehe et al., 2013).

Practical Challenge 2—Fluid homogeneity The density of carbonyl iron powder is 7.86 g/cm^3^ (Peng et al., 2024), whereas the density of most carrier fluids ranges between 0.8 and 1.0 g/cm^3^ (Eshgarf et al., 2022). This significant density difference inevitably leads to sedimentation under body forces. Sedimented MRF can have a reduced yield stress of up to 30% (Kikuchi et al., 2017), meaning that the torque for a given magnetic field can be reduced, causing variable performance depending on the state of the fluid. The extended periods for which an aircraft can remain grounded in the aerospace industry make this issue particularly problematic. Many studies have been conducted on various MRF formulations to reduce sedimentation (Choi, 2022), but gravity always wins and sedimentation will inevitably occur over extended periods of time. Circulation in the shear interface can help remix the fluid and mitigate the effects of sedimentation.

A mitigation of these two challenges is to force MRF circulation in the shear interfaces of the clutch to distribute wearing on the entire fluid volume within the clutch while reducing temperature; and to mitigate the impact of sedimentation by continuously remixing the fluid. Experimental investigations in simulated laboratory conditions show that increasing the fluid volume in a MR clutch and ensuring mixing of the fluid between the drums through an MRF circulation device extends the actuator assembly’s lifetime (Desrosiers et al., 2013; Pilon et al., 2020; Smith et al., 2007). However, there is no readily available MRF pumping solution that can meet aerospace reliability requirements. It is also worth mentioning that pumping plays a central role in any MR flow mode application. While the literature primarily focuses on the design, control, and optimization of flow mode devices such as MR valves (Abd Fatah et al., 2015; Hu and Wereley, 2003; Nguyen et al., 2007, 2009; Wereley and Pang, 1998; Yoo and Wereley, 2002), there has been limited focus on the pump itself.

### 1.2. Background and literature

This section presents an overview of applicable solutions for circulating MRF in clutches, a process that is highly challenging due to the fluid’s high viscosity (0.2–0.3 Pa·s; Eshgarf et al., 2022) and the large concentration of micron-sized particles, which can reach up to 45% V/V (Eshgarf et al., 2022). It is followed by an introduction on pump theory, focusing on dimensionless numbers and scaling laws.

#### 1.2.1. MRF pump

Rabinow patented the idea of pumping MRF into a clutch (Rabinow, 1951). The concept aimed to use an external pump to enhance the homogeneity of the fluid. However, this approach would increase the complexity and weight of an actuator system by adding a non-integrated external pump. For aerospace applications, pumps must be lightweight and integrated directly in clutches, ideally powered by the same input shaft of the clutch.

An integrated circulation mechanism based on screw pumps was proposed by Winslow (1959). Spiral grooves were added to the usual smooth drum of a clutch, directly in the shear interface. Different groove geometries have been used (Plante et al., 2018; Usoro et al., 2001). The aim was to create circulation to extend the clutch’s lifespan and reduce hotspots.

Pilon et al. (2020) studied the fluids mechanics of MRF circulation in screw pumps using a single-drum test clutch on the drum surfaces. A maximum flow of 25 mL/min was measured. The work showed that the pumping mechanics is not mechanical such as in a classic screw pump as anticipated by Winslow, but magnetic. Indeed, there is no pumping without application of a magnetic field. “Magnetic” screw threads are actually formed by agglomerated carbonyl-iron particles outside the grooves machined on the drums where the magnetic field is stronger.

Since magnetic screw pumps only work in the presence of a magnetic field, and preferably a strong one, the potential of theses pumps to uniformize the distribution of particles in the shear interface is limited to highly loaded operating conditions. MRF circulation must be independent of the magnetic field or clutch loading.

Looking at conventional pump designs, the most logical approach to pump highly viscous fluids or slurries such as MRF is to use positive displacement pumps (PDPs). However, integrating a PDP within an aerospace MR clutch defeats the purpose due to metal-to-metal contacts.

**Figure 2.**
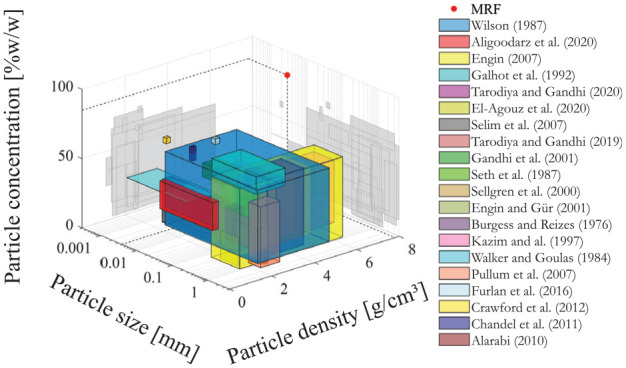
Slurry properties of experimental centrifugal pump study in literature.

Turbomachinery pumping is the only conventional approach to pump MR fluid without metal-metal contacts. Many studies have been conducted on the handling of viscous slurries by centrifugal pumps (EL-Agouz et al., 2020; Alarabi, 2010; Aligoodarz et al., 2020; Burgess and Reizes, 1976; Chandel et al., 2011; Crawford et al., 2012; Engin, 2007; Engin and Gur, 2001; Furlan et al., 2016; Gahlot et al., 1992; Gandhi et al., 2001; Kazim et al., 1997; Pullum et al., 2007; Selim et al., 2007; Sellgren et al., 2000; Sheth et al., 1987; Tarodiya and Gandhi, 2019, 2020; Walker and Goulas, 1984; Wilson, 1987). However, MR fluid properties are far away from any fluid tested in the literature as showed in the survey of Figure 2. Therefore, discussing the potential integration a centrifugal pump in a MR clutch, it is essential to gain an understanding of the physics of MRF pumping using a centrifugal pump.

#### 1.2.2. Centrifugal pump scaling laws

Dimensionless numbers play a crucial role in characterizing pump performance, notably the capacity coefficient and the head coefficient (White, 2011).

The capacity coefficient, denoted as $C_{Q}$ represents the flow rate in relation to the rotational speed and diameter of the impeller. It is defined by the following equation:

(1) $$C_{Q}=\frac{Q}{ND^{3}}$$

where Q is the volumetric flow rate, N is the rotational speed, and D is the impeller diameter. Similarly, the head coefficient, denoted as $C_{H}$ provides a measure of the pump’s capability to generate pressure. It is defined as:

(2) $$C_{H}=\frac{\mathrm{Hg}}{N^{2}D^{2}}$$

where H is the head and g is the acceleration due to gravity. The relationship between head and pressure is given by:

(3) P=ρgH

where P is the pressure and ρ is the fluid density.

Scaling laws derived from these coefficients predict how a pump’s performance changes when its size or rotational speed are modified. For instance:

- The flow rate Q varies linearly with the rotational speed N and with the cube of the impeller diameter D :
$$Q\propto ND^{3}$$
- The head H varies with the square of the rotational speed N and with the square of the impeller diameter D :
$$H\propto N^{2}D^{2}$$
- The pressure P generated by the pump, based on the head, varies similarly:
$$P\propto \rho N^{2}D^{2}$$

Classical scaling laws are derived based on the assumption of a single-phase homogeneous fluid. However, at high particle concentrations, the fluid exhibits complex rheological behavior driven by intense particle interactions. In pumping systems, the induced g-field from the impeller’s rotation may lead to uneven fluid density, turbulence, and particle agglomerations. These phenomena can significantly alter pump performance and challenge the validity of standard scaling laws.

### 1.3. Approach

Scaling laws are valuable as predictive tools to design pumps with the desired performance. These laws provide a means of optimizing the pump design without requiring extensive resources or repeated experimental tests. However, due to the high particle concentration in MRFs, it is necessary to validate these laws experimentally to ensure their reliability in this specific context.

This paper has two objectives of: (1) determining whether MRF pumping adheres to the usual centrifugal pump scaling laws despite high particle concentrations, and (2) assessing the feasibility of centrifugal pumping in a commercial clutch.

To achieve these objectives, the performance of centrifugal pump prototypes with MRF is characterized experimentally. Then, the validity of the scaling laws when speed and size vary is verified. Finally, the feasibility of using a centrifugal pump is assessed by pairing a pump design to a theoretical hydraulic model of a real commercial clutch to identify the operating point, simultaneously determining the corresponding flow rate per shear interface of the clutch.

The article is divided in fives sections which present the clutch hydraulic system modeling, the experimental pump and test bench, the test protocols, results, and conclusion.

## 2. Hydraulic system modeling of the shear interface in a commercial MR clutch

**Figure 3.**
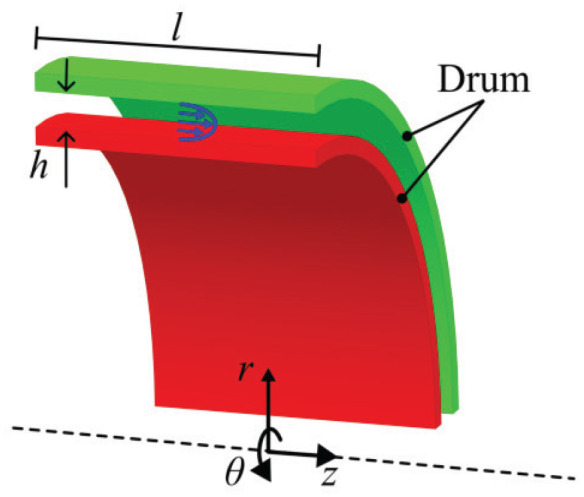
MRF flow in the shear interface of a drum-type MR clutch. The blue arrows show the velocity profile.

This section presents the theoretical model of the hydraulic system of the shear interface in a MR clutch. Figure 3 shows the MRF flow in the shear interface of a drum-type MR clutch.

Two assumptions are made: (1) the flow is viscous, and (2) the Couette flow caused by the drum’s speed differential does not affect the flow in the *z*-direction. Given the large drum radius compared to the fluid gap *h*, the problem is simplified by converting from cylindrical to Cartesian coordinates. The flow can then be approximated as a pressure-driven flow between two flat plates. The following equation represents the resulting pressure versus flow relationship:

(4) $$Q=\frac{h^{3}b}{12\mu }\frac{\Delta P}{l}$$

where *h* the fluid gap between two drums, *b* is the sum of the circumferences at the mean radius between two drums, ΔP is the pressure generated by the pump, *l* is the length of the drum, and μ the viscosity of the fluid.

The geometry of a commercial drum-type MR clutch from Exonetik, is used to set a targeted pressure versus flow rate curve. Typical drum-type MR clutches have multiple drums (six for the clutch in the study case), creating several shear interfaces through which the fluid circulates. In MR clutch with screw pump, grooves are machined on each drum, acting like individual pumps for each shear interface. Since only one centrifugal would be used to create flow in all six shear interfaces, the flow rate is express as a flow per shear interface ( $\bar{Q}$ ) for fair comparison. The result is shown in the following equation:

(5) $$P=0.1\bar{Q}$$

This model will later be compared to centrifugal pump curves obtained experimentally to find the operating point of the pump and the resulting flow rate per shear interface.

## 3. Experimental test bed design

### 3.1. Pump design

**Figure 4.**
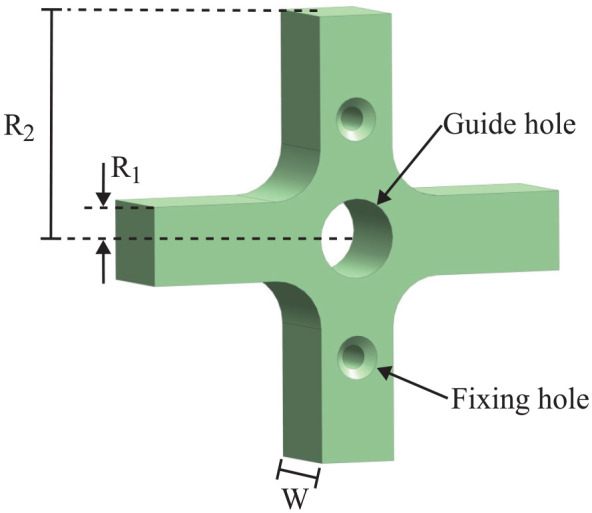
Cross impeller design.

**Figure 5.**
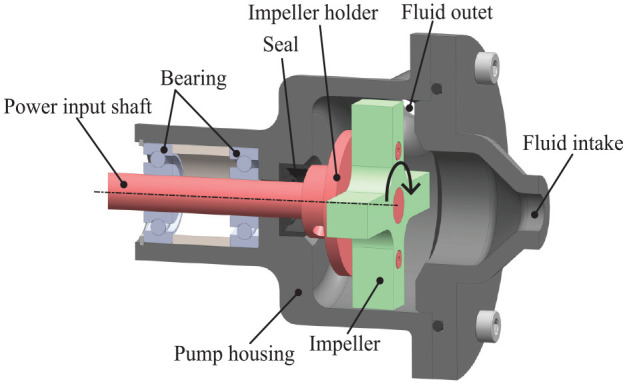
MRF centrifugal pump assembly side cross section. Red and green parts indicate rotating components.

**Figure 6.**
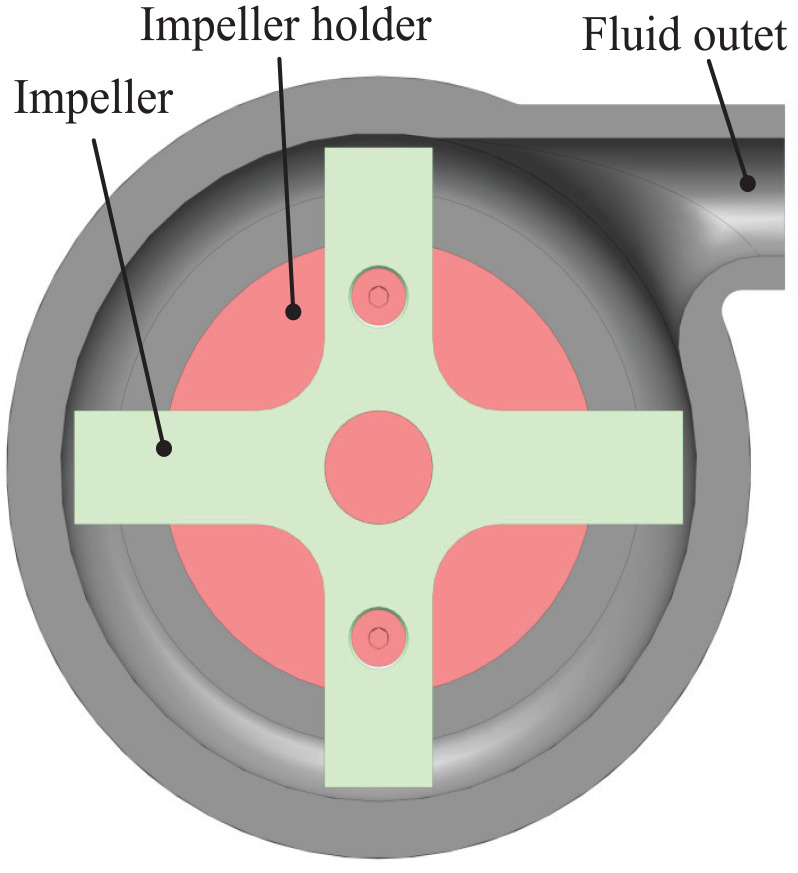
MRF centrifugal pump output face cross section.

A basic cross impeller (Figure 4) is selected for its robustness and potential ease of manufacturing, despite not being the most efficient design. The same pump design, scaled to two different sizes, was tested, with the impeller dimensions detailed in Table 1. Both the impeller and the housing were proportionally scaled to respect the criterion of geometric similarity. The gaps between the impeller tips and the housings are 0.2 mm for D1 and 0.4 mm for D2. However, readers should note that the fabrication precision is ±0.1 mm. The pump intake aligns concentrically with the impeller’s rotation axis, while the output is tangent to the impeller, as illustrated in Figure 5. The pump has no volute and a plain tangential outlet for simplicity (Figure 6). The seal used is an NBR skeleton oil seal. Both impeller and housing of the pump are 3D printed in PLA with a 100% infill.

**Table 1.** Impeller specifications.

Impeller	*R*_1_ (mm)	*R*_2_ (mm)	*W* (mm)	Blade number
D1	2	11.5	5	4
D2	4	23	10	4

### 3.2. Test bench and sensors

**Figure 7.**
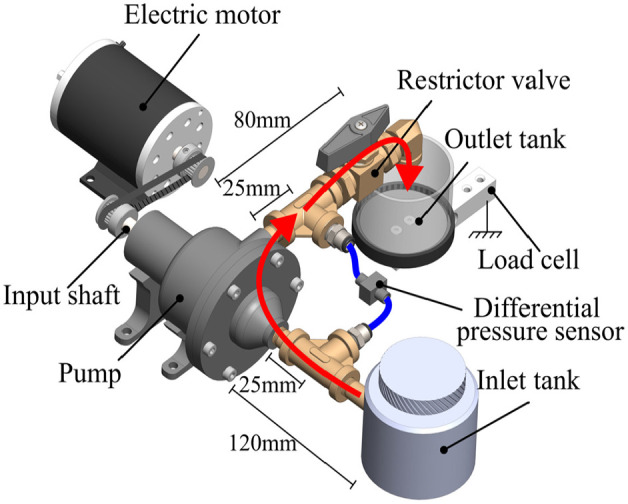
Pump characterization test bed. The red arrows represent the flow path.

The pump characterization test setup is depicted in Figure 7. The pump is powered by a 500W DC electric motor (MY-1020) with a 1:1 strap ratio. Motor speed control is managed through an Arduino MEGA 2560, which sends the speed command to an AMC 120A10 drive. Flow rate measurement follows open-loop methodology (Pilon et al., 2020). The output flow rate is directed into a reservoir mounted on a 1 kg beam load cell, and the flow rate is determined by deriving the force signal from the load cell as given in:

(6) $$Q=\frac{1}{\rho }\frac{\mathrm{dm}}{\mathrm{dt}}$$

where Q is the flow rate, ρ the density of the fluid, m the mass read by the load cell, and t , the time. Due to low flow rates, momentum variations can be neglected. The open-loop method offers advantages such as low cost and absence of flow restriction, but limits test duration.

The total head generated by the pump is measured with a 5 psi differential pressure sensor (Omega PX26-005DV) and an amplifier (Omega DRF-LC). Pressure readings are taken at the pump outlet and just before the intake as illustrated in Figure 7. All pipes and fittings are 1/8″ NPT standard except between the inlet tank and the T fitting from the pressure sensor. In that section, 1/4″ NPT was used to minimize risks of pump stalling due to excessive inlet head losses. A ball valve is installed after the pressure sensor at the pump outlet as a flow restriction and way of varying the pump’s operating point. A Hall effect speed sensor (KY-003) is employed to measure rotation speed. Data acquisition and control are managed by the Arduino platform.

## 4. Test protocols

A linear speed-up ramp of 2 s is used for each test to ensure repeatability and prevent pressure bursts.

Before each test, the MRF is remixed using a pneumatic paint shaker for 5 min to ensure an even distribution of particles in the fluid. Additionally, the pump and piping system are bled by pressurizing the MRF tank until the exit flow is free of air bubbles. All tests were conducted at a constant ambient temperature of 23°C, with no additional cooling applied to the experimental setup. The properties of the MRF used for all tests in this study are shown in Table 2.

**Table 2.** MRF properties.

Properties	MRF
Density	3.49 g/cm^3^
Particles concentration	40%V/V
Particles average size	3.8 μ m
Viscosity at 23°C	0.22 Pa·s

Three different types of tests were performed to characterize the pump: flow rate as a function of time, maximum pump pressure as a function of RPM, and pump curve characterization. The flow and maximum pressure tests are conducted to assess the pump’s capacity and head coefficient individually, while the pump curve characterization evaluates both parameters simultaneously.

### 4.1. Flow test methodology

The first characterization test measures the flow rate as a function of time. The dimensions of the two pump tested are shown is Table 1. Both pumps have been tested up to 3000 rpm while the restrictor was fully open.

### 4.2. Maximum pressure test methodology

The restrictior valve of Figure 7 is completely closed to measure maximum pressure. Tests were conducted from 1000 to 3000 rpm in increments of 250 rpm. The maximum rotation speed is limited to 2250 rpm due to reaching the upper limit of the pressure sensor for the D2 impeller. The mean of the pressure signal is calculated over 20 s once the steady state of the pump is reached after the speed-up ramp of the electric motor.

### 4.3. Pump curve test methodology

A pump that is head pressure as a function of the flow rate is traced by starting the pump with the restrictor valve wide open and gradually closing it. Delay between pressure measurement signal and flow measurement are neglected, as the manual closing of the valve has a much longer timescale than the data acquisition rate.

## 5. Results

### 5.1. Flow characterization

**Figure 8.**
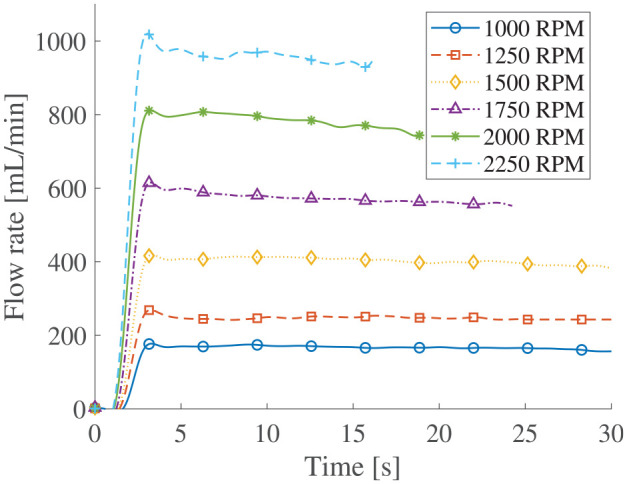
Flow rate function of time for D2 impeller with MRF.

The flow rates function of time at different rotation speeds for impellers D2 with MRF are shown in Figure 8. The tests at 1750, 1500, and 2250 rpm were stopped before 30 s due to flow rate open loop measurement method. The flow rates are relatively constant but tended to drop. A small part of the flow drop can be associated with the minor fluid height change in the inlet tank. Small oscillations are observed due to the vibration of the load cell induced by the electric motor. The impact of the momentum variation can be observed at around 2.5 s where the flow rate reaches a peak and is overvalued for a short period of time.

**Figure 9.**
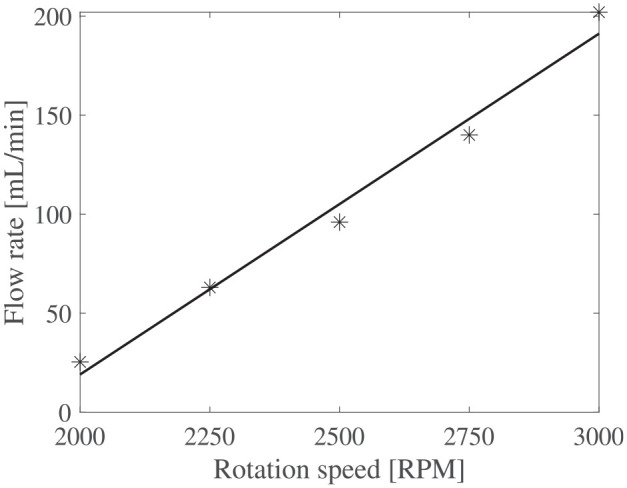
Flow rate function of rotation speed for D1 impeller.

**Figure 10.**
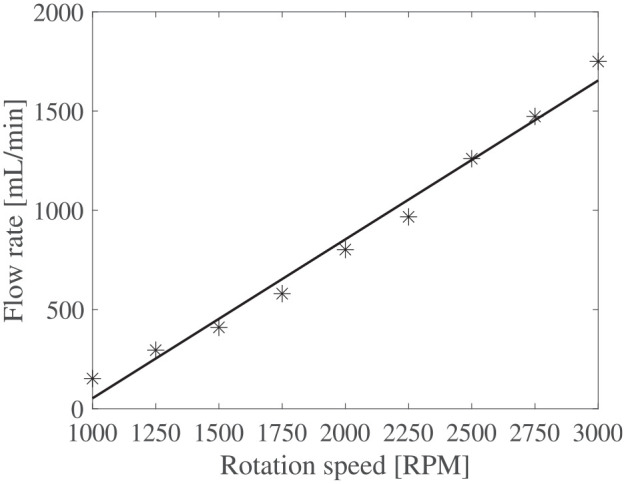
Flow rate function of rotation speed for D2 impeller.

The flow rates versus rotation speed for both impeller D1 and D2 are shown in Figures 9 and 10. These results are the average flow rate during the steady state of the flow function of time test. Usual scaling laws of centrifugal pumps state that the flow rate should vary linearly with the rotation speed. Strong correlation coefficients $R^{2}$ of 0.98 are observed for both impeller D1 and D2. The results suggest that the high particle concentration in MRF does not affect the capacity coefficient with respect to flow rate changes due to rotation speed.

**Figure 11.**
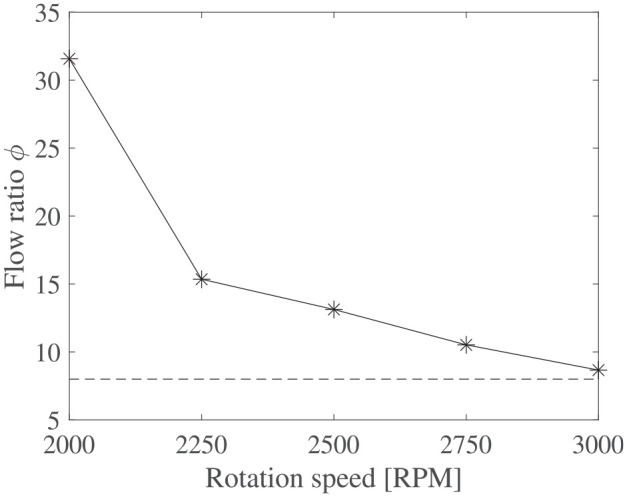
Experimental flow ratio between D1 and D2 impeller function of rotation speed.

Now, regarding impeller size, for a given rotation speed, considering that the D2 impeller is twice the size of D1, the flow rate of D2 should be eight times higher than D1 when scaling according to capacity coefficients:

(7) $$\phi =\frac{Q_{2}}{Q_{1}}\propto \frac{d_{2}^{3}}{d_{1}^{3}}$$

with ϕ the volumetric flow rate ratio, $Q_{i}$ the flow rate for the impeller of diameter $d_{i}$ . The flow ratio ϕ function of the rotational speed between 2000 and 3000 rpm is shown in Figure 11. As RPM increases, the flow ratio ϕ gradually tends to the theoretical value. At lower rotation speed, where the viscous effect have greater impact in the pump performance, the roughness, and clearance ratio of the D2 impeller are lower, resulting in a suspected higher pump efficiency (Wilson et al., 2006).

### 5.2. Maximum pressure characterization

**Figure 12.**
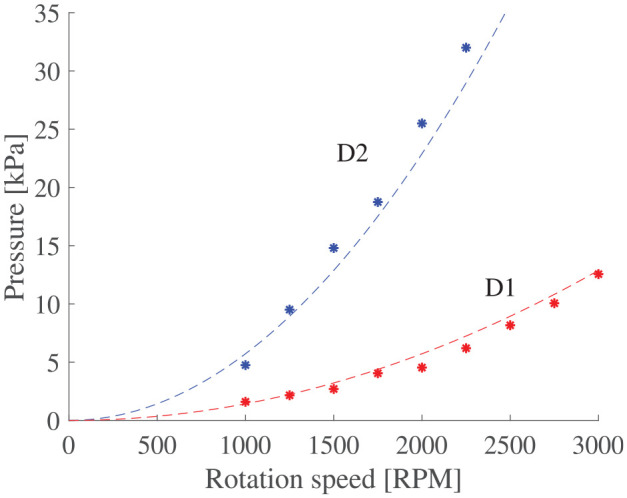
Static pressure from cross impeller centrifugal pump.

Figure 12 shows the differential pressure as a function of pump RPM. The experimental results are compared to the theoretical maximum pressure obtain from Euler turbomachinery equations (White, 2011) with an efficiency factor $n_{\mathrm{eff}}$ :

(8) $$P_{\max}=\eta_{\mathrm{eff}}\rho \omega^{2}R_{1}^{2}$$

where ω is the rotational speed in rad/s.

Theoretical model predictions of Figure 12 are obtained with a value of 27% for $n_{\mathrm{eff}}$ , offering the best possible fit with a mean relative error of 12.6% between the model and the experimental data for both impeller. In comparison, for a centrifugal pump using standard fluid, the efficiency $n_{\mathrm{eff}}$ is about 60% in the literature (White, 2011). It can be concluded that high particle concentration does impact the capability of a centrifugal pump to generate no-flow pressure. High particles concentration can increase friction in the fluid and thus, reduce the efficiency factor.

Despite low efficiency factor, the pressure varies with the square of the rotational speed and the square of the impeller diameter, just like the head coefficient scaling law of equation (2).

### 5.3. Pump curve characterization

**Figure 13.**
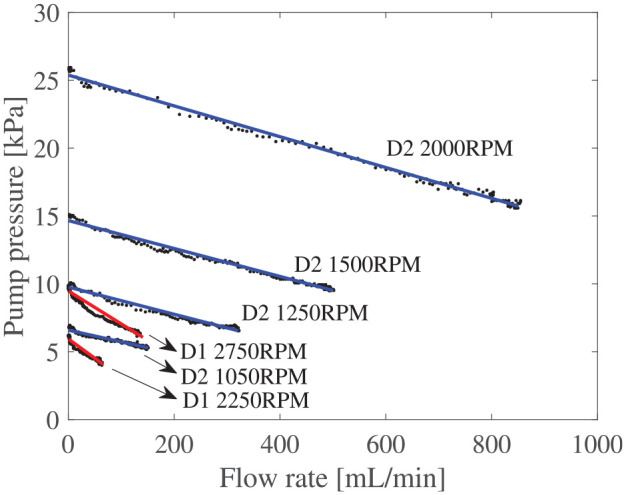
Pump curve for D1 and D2 impeller.

The pump curves with MRF for different rotation speeds are presented in Figure 13. The pump curve characterization of the D1 impeller was done at higher rotation speed (2750 and 2250 rpm) than the D2 impeller (1100, 1250, 1500, and 2000 rpm) as smaller clutches are expected to spin faster in future applications.

For the D1 impeller at 2750 rpm, the maximum flow rate is 136 mL/min and a maximum pressure of 9.9 kPa. At 2000 rpm, the D2 impeller have a maximum flow rate of 850 mL/min and a maximum pressure of 25.5 kPa.

### 5.4. Dimensionless pump curve

**Figure 14.**
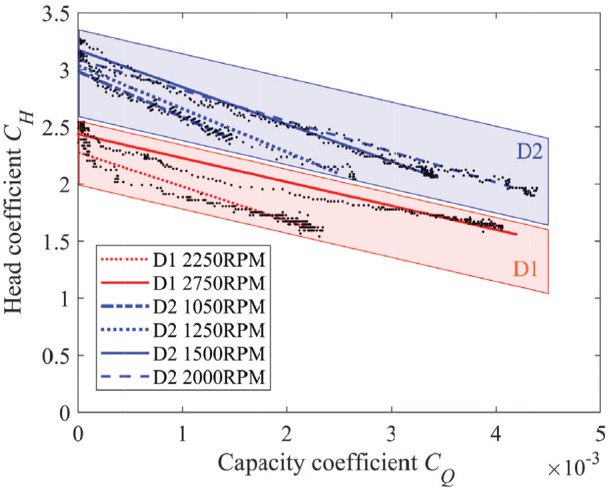
Dimensionless pump curve for cross impeller centrifugal pump with MRF.

The pressure-flow curves in Figure 13 can be non-dimensionalized by plotting the head coefficient against the capacity coefficient, as shown in Figure 14. Given the same pump design, these curves should overlap regardless of impeller size or rotational speed. However, the results show a noticeable difference between the D1 and D2 curves. The D2 impeller demonstrates better performance than the D1 impeller. Here, performance is defined as the pump’s ability to achieve a high capacity coefficient for a given head coefficient. This performance difference is most likely due to reduced impeller efficiency associated with smaller scale, as observed by Wilson et al. (2006). The exact impeller efficiency was not measured and is left for future work, due to the complexity arising from the unknown efficiency of test bed components.

Nevertheless, both curves for a given impeller are similar, confirming that scaling laws can accurately predict performance changes with varying rotation speeds for a given impeller. Building on this observation, the next section presents a semi-empirical model to predict pump curves at different speeds for the D2 impeller.

In summary, while dimensionless pump curves can provide a rough estimate of pumping performance across different operating speeds for a given impeller size, they are not precise enough for predicting the performance of pumps at various scales when pumping MRF.

### 5.5. Semi-empirical model

A dataset from D2 impeller including the static pressure measurement for different rotation speeds and pressure-flow curves at 1050, 1500, and 2000 rpm from Figure 13 is used to generate a semi-empirical pumping model (equation (9)). The pressure-flow curve at 1250 rpm is not used for the model generation and is instead used as a tool for experimental validation of the model.

(9) $$P{\left(Q\right)}_{D_{2}}=\eta_{\mathrm{eff}}\omega^{2}\rho R_{2,D2}^{2}-0.0101Q$$

**Figure 15.**
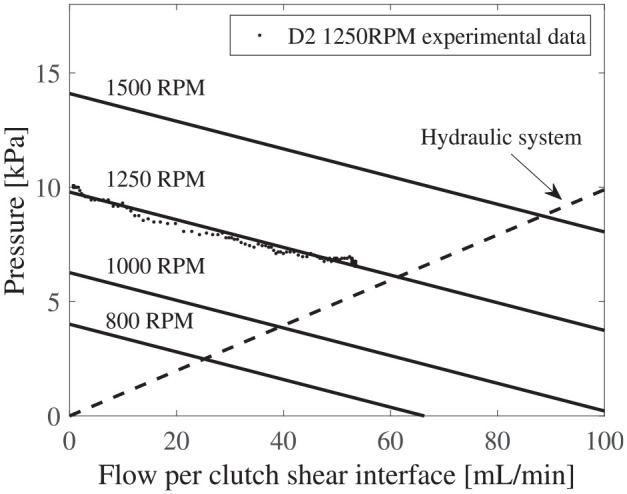
Operating point of centrifugal pump with D2 impeller in a commercial MR clutch.

Figure 15 shows the pump curves obtained from the model and the experimental data of the pressure-flow curve at 1250 rpm. The agreement between the semi-empirical model and the independent experimental data is strong, with a relative error of 2%, thereby validating the proposed model.

### 5.6. Operating point in commercial clutch

The hydraulic system model of a clutch (Section 2) and pump curve obtained from the semi-empirical model are plotted together to determine the operating point and consequent flow rate through a shear interface.

Figure 15 shows the results for the D2 impeller, which was designed to match the dimensions of the commercial MR clutch used in the hydraulic model in section 2.

The pressure range produced by the pump enables high flow rates in the shear interface. At 800 rpm, the flow rate is 24.8 mL/min per shear interface, matching the maximum flow rate achieved by Pilon with the magnetic screw pump. Increasing the rotation speed to 1500 rpm boosts the flow rate to 90 mL/min per shear interface.

## 6. Conclusion

This work studied the potential of centrifugal pumping MRF. The objectives of the paper were to determine whether MRF pumping adheres to the usual centrifugal pump scaling laws despite high particle concentrations and to assess the feasibility of using a centrifugal pump in a clutch by estimating the flow rate that would be obtained in a commercial clutch.

All objectives have been achieved by experimentally testing centrifugal pump prototypes with MRF. Two sizes of cross impeller centrifugal pump have been designed and bench-tested.

Usual centrifugal pump scaling laws accurately predict performance changes with varying rotation speeds, even with a fluid composed of 40% V/V of ferromagnetic microparticles. However, they are less reliable for predicting performance variations when the impeller diameter is scaled, here by a factor of two, because of significant changes in pumping performance.

A semi-empirical model is proposed to obtain a pump curve for different rotation speeds for each impeller tested. At 800 rpm, the theoretical flow produced per shear interface in a commercial MR clutch is 25 mL/min which is the maximum flow reported in the literature. The centrifugal pump has the potential to create a lot of high flow while being independent of the magnetic field or clutch loading. At 1500 rpm, the D2 impeller could produces 90 mL/min per shear interface.

Further investigations should validate the effect of flow rate on fluid mixing. The hydraulic resistance of the system with magnetic field also need to be studied. In addition, modeling two-phase flow computational fluid dynamics at a pump-particle interaction level would be useful to better understand pump performance with sedimented fluid (non-homogenous). Future work should also explore the influence of temperature variations and different MRF concentrations on pump behavior.
